# Supplementary material for: Nafcillin-Loaded Photocrosslinkable Nanocomposite Hydrogels for Biomedical Applications
Source: Pharmaceutics. 2023 May 24;15(6):1588. doi: 10.3390/pharmaceutics15061588 (PMC10304380; doi:10.3390/pharmaceutics15061588)
Supplement: Supplementary file 1 [file pharmaceutics-15-01588-s001.zip › pharmaceutics-2398425-supplementary.pdf]

Supplementary material

Nafcillin-Loaded Photocrosslinkable Nanocomposite Hydrogels for Biomedical Applications

Gabriela Toader [1,†], Ionela Alice Podaru [1,2,†], Edina Rusen [2,*], Aurel Diacon [1,2], Raluca Elena Ginghina [3], Mioara Alexandru [4,*], Florina Lucica Zorila [4,5], Ana Mihaela Gavrila [6], Bogdan Trica [6], Traian Rotariu [1] and Mariana Ionita [2,7]

[1] Military Technical Academy, "Ferdinand I", 39-49 G. Cosbuc Blvd., 050141 Bucharest, Romania; nitagabriela.t@gmail.com (G.T.); podaru.alice04@gmail.com (I.A.P.); aurel_diacon@yahoo.com (A.D.); trotariu@mta.ro (T.R.)
[2] Faculty of Chemical Engineering and Biotechnologies, University Politehnica of Bucharest, 1–7 Gh. Polizu Street, 011061 Bucharest, Romania; mariana.ionita@polimi.it
[3] Research and Innovation Centre for CBRN Defense and Ecology, 225 Şos. Olteniţei, 041327 Bucharest, Romania; raluca.ginghina@nbce.ro
[4] Microbiology Laboratory, Horia Hulubei National Institute for R&D in Physics and Nuclear Engineering, 30 Reactorului St., 077125 Magurele, Romania; florina.zorila@nipne.ro
[5] Department of Genetics, Faculty of Biology, University of Bucharest, 91-95 Splaiul Indepentenei, 050095 Bucharest, Romania
[6] National Institute of Research, Development for Chemistry and Petrochemistry, 202 Splaiul Independentei, 060041 Bucharest, Romania; ana.gavrila@icechim.ro (A.M.G.); bogdan.trica@icechim.ro (B.T.)
[7] eBio-Hub Research Centre, University Politehnica of Bucharest-Campus, Iuliu Maniu 6, 061344 Bucharest, Romania
* Correspondence: edina.rusen@upb.ro (E.R.); mioara.alexandru@nipne.ro (M.A.)
† These authors contributed equally to this work.

Contents

Figure S1. FT-IR analysis for the synthesized hydrogels, TiO2, ZnO and monomers (NVP, TEGDVE) 2
Figure S2. SEM image and EDX mapping of lyophilized P1 3
Figure S3. SEM image and EDX mapping of lyophilized P1-TiO_2 4
Figure S4. SEM image and EDX mapping of lyophilized P1-ZnO 5
Figure S5. SEM image and EDX mapping of lyophilized P2 6
Figure S6. SEM image and EDX mapping of lyophilized P2-TiO_2 7
Figure S7. SEM image and EDX mapping of lyophilized P2-ZnO 8
Figure S8. SEM images of lyophilized samples 9
Figure S9. Exemplification of equilibrium - swollen nanocomposite hydrogel films 9
Figure S10. Nafcillin loading efficiency in the hydrogels 10
Figure S11. Nafcillin release - data fitting for sample P1 10
Figure S12. Nafcillin release - data fitting for sample P2 11
Figure S13. Nafcillin release - data fitting for sample P2.1 (P2 – TiO_2) 12
Figure S14. Nafcillin release - data fitting for sample P2.2 (P2 - ZnO) 13

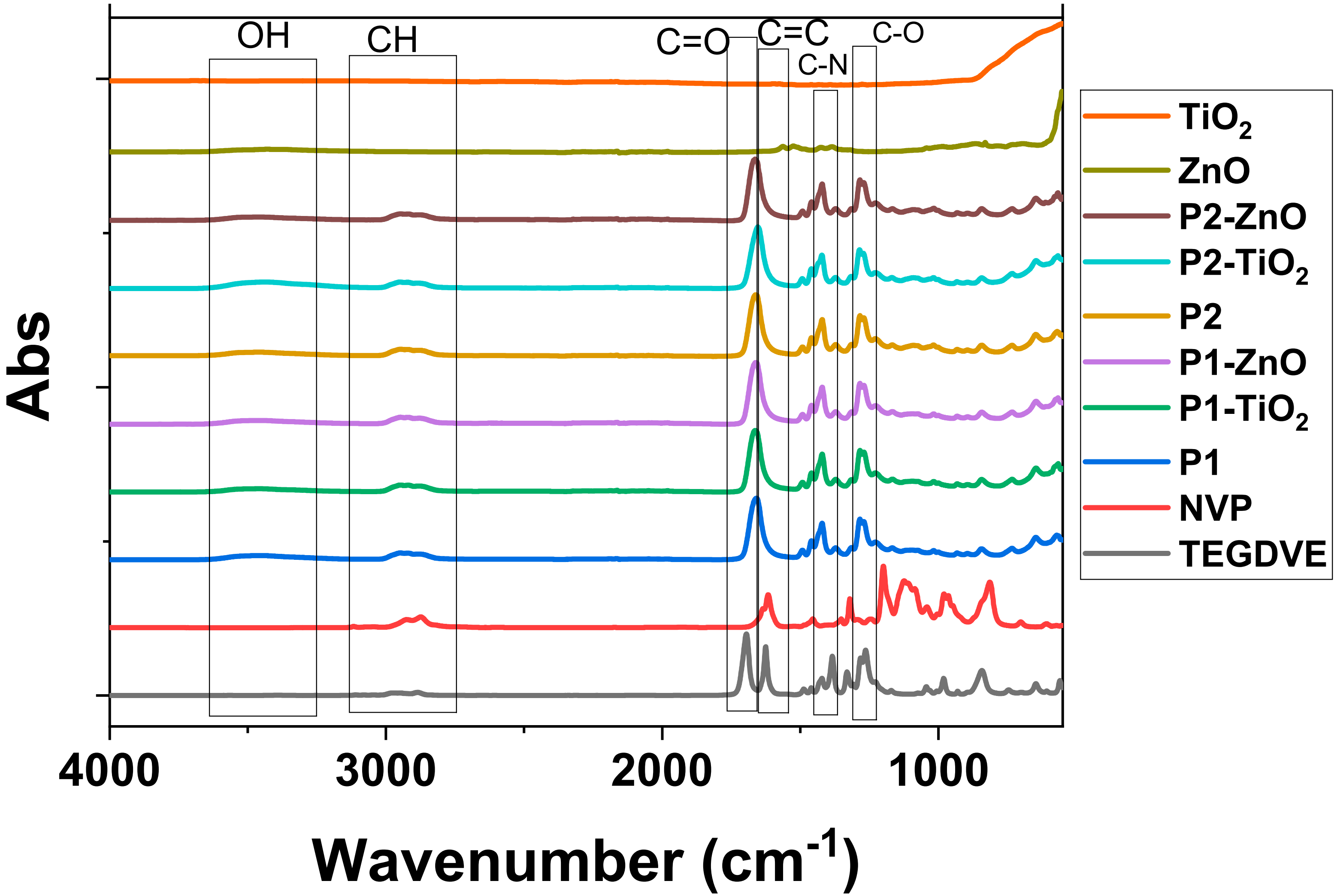

Figure S1. FT-IR analysis for the synthesized hydrogels, TiO2, ZnO and monomers (NVP, TEGDVE)

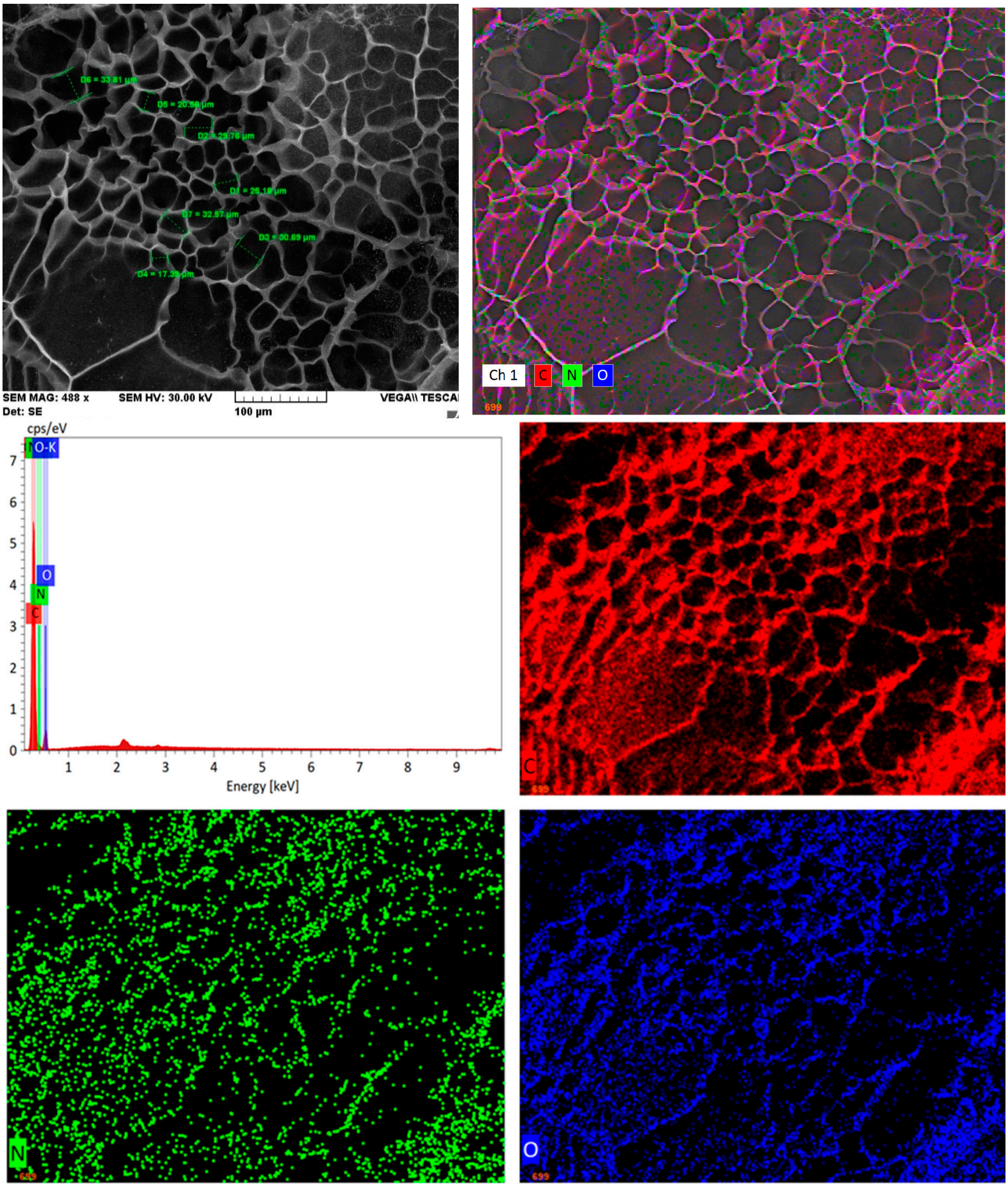

Figure S2. SEM image and EDX mapping of lyophilized P1

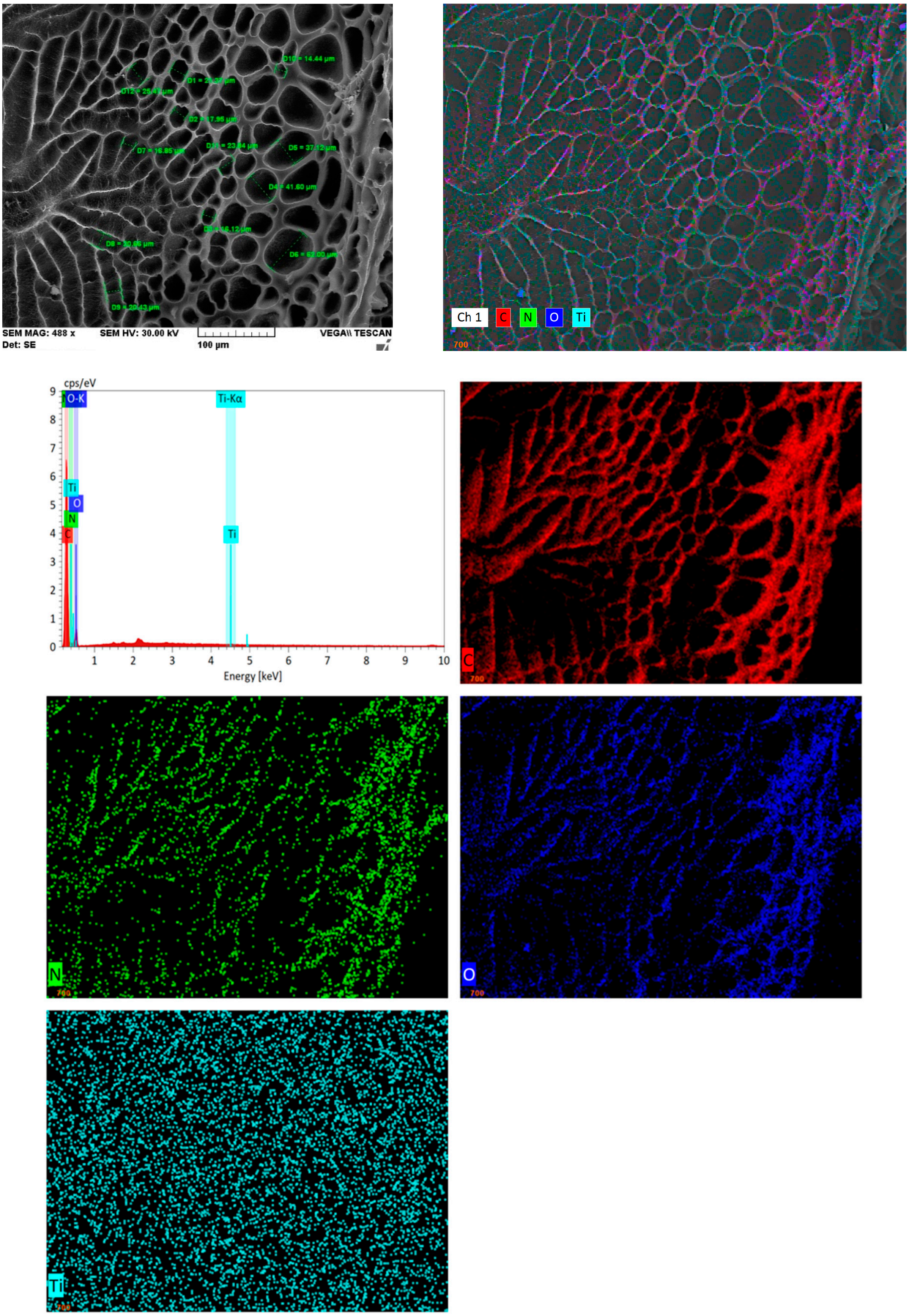

Figure S3. SEM image and EDX mapping of lyophilized P1-TiO_2

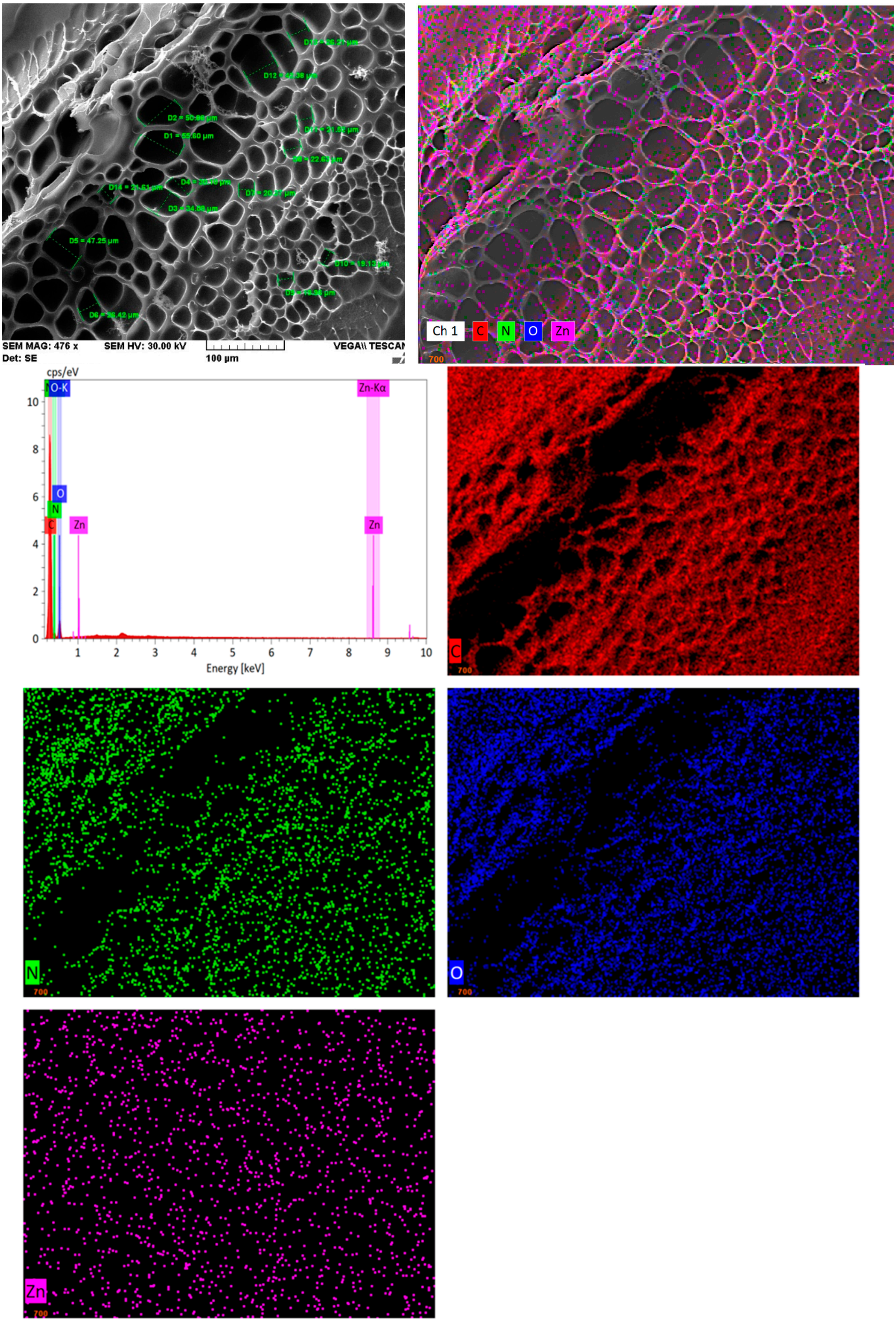

Figure S4. SEM image and EDX mapping of lyophilized P1-ZnO

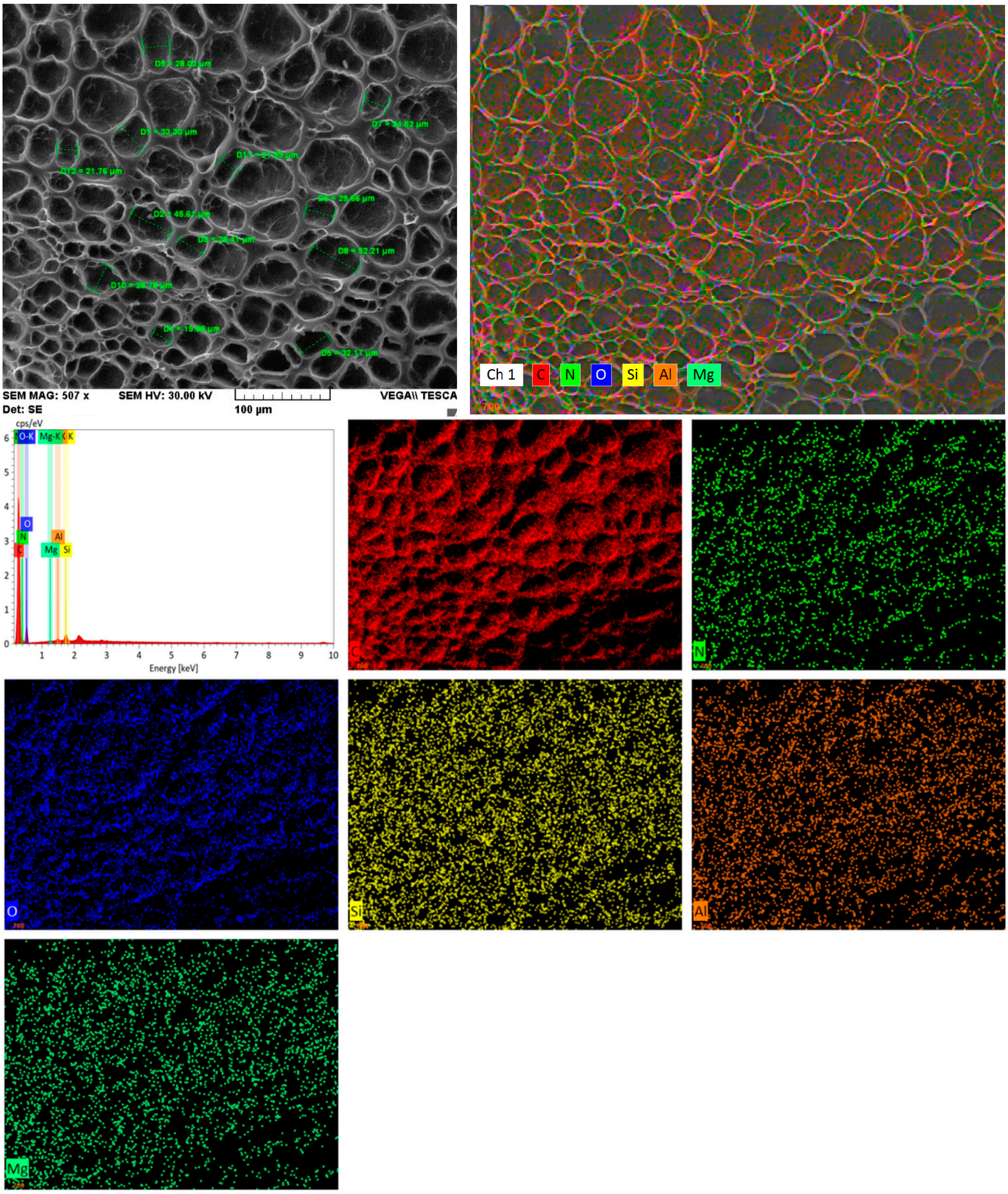

Figure S5. SEM image and EDX mapping of lyophilized P2

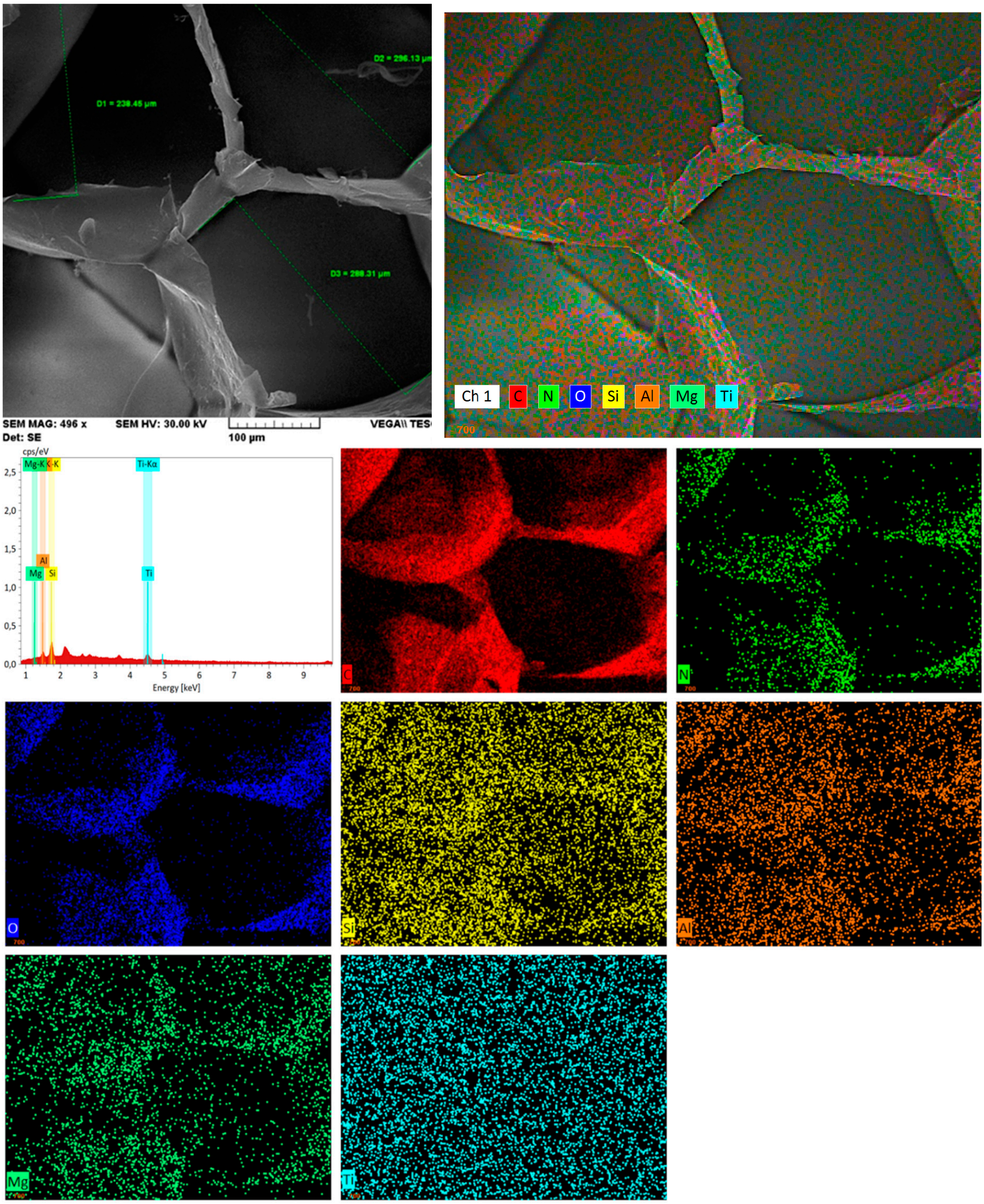

Figure S6. SEM image and EDX mapping of lyophilized P2-TiO_2

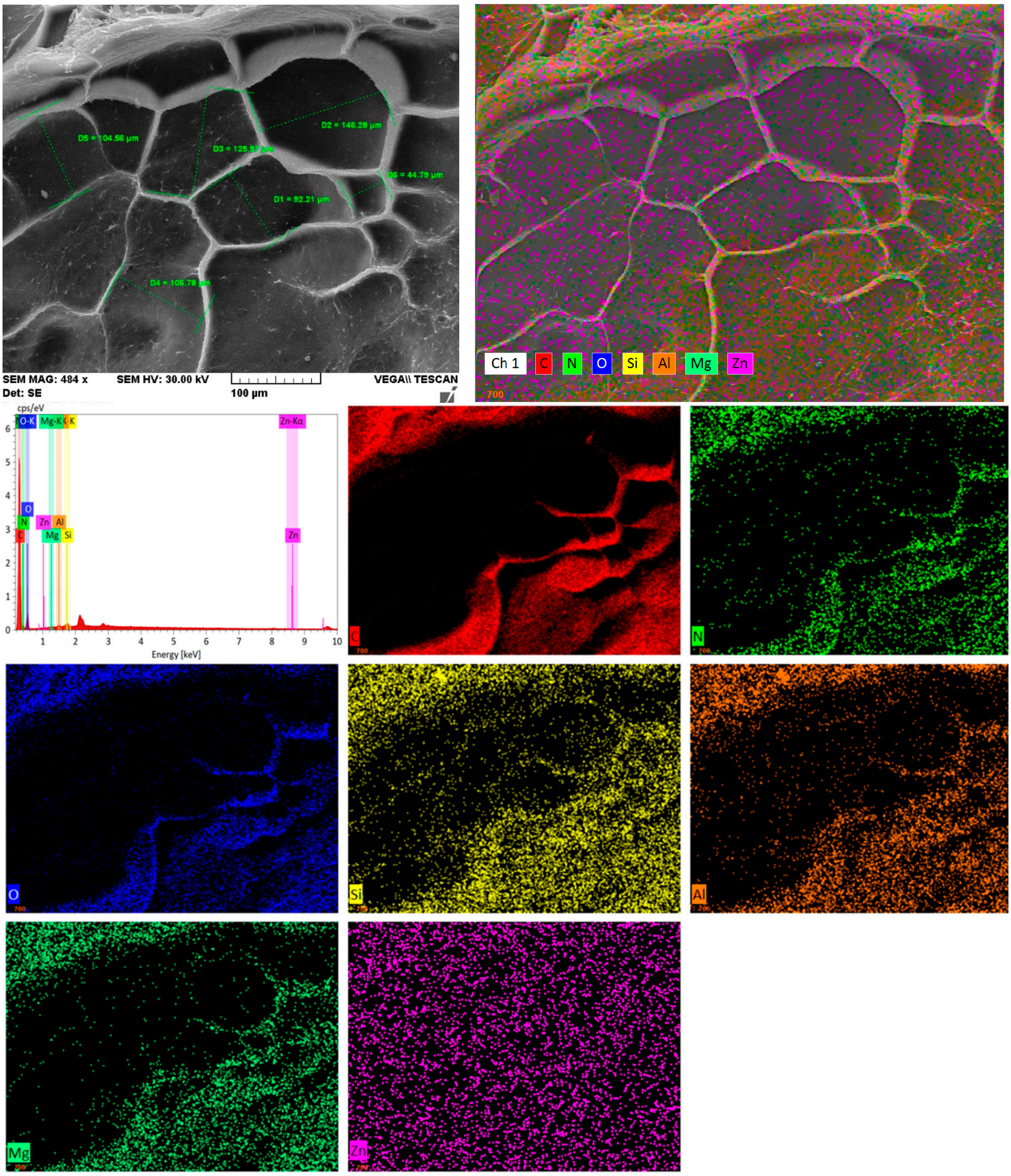

Figure S7. SEM image and EDX mapping of lyophilized P2-ZnO

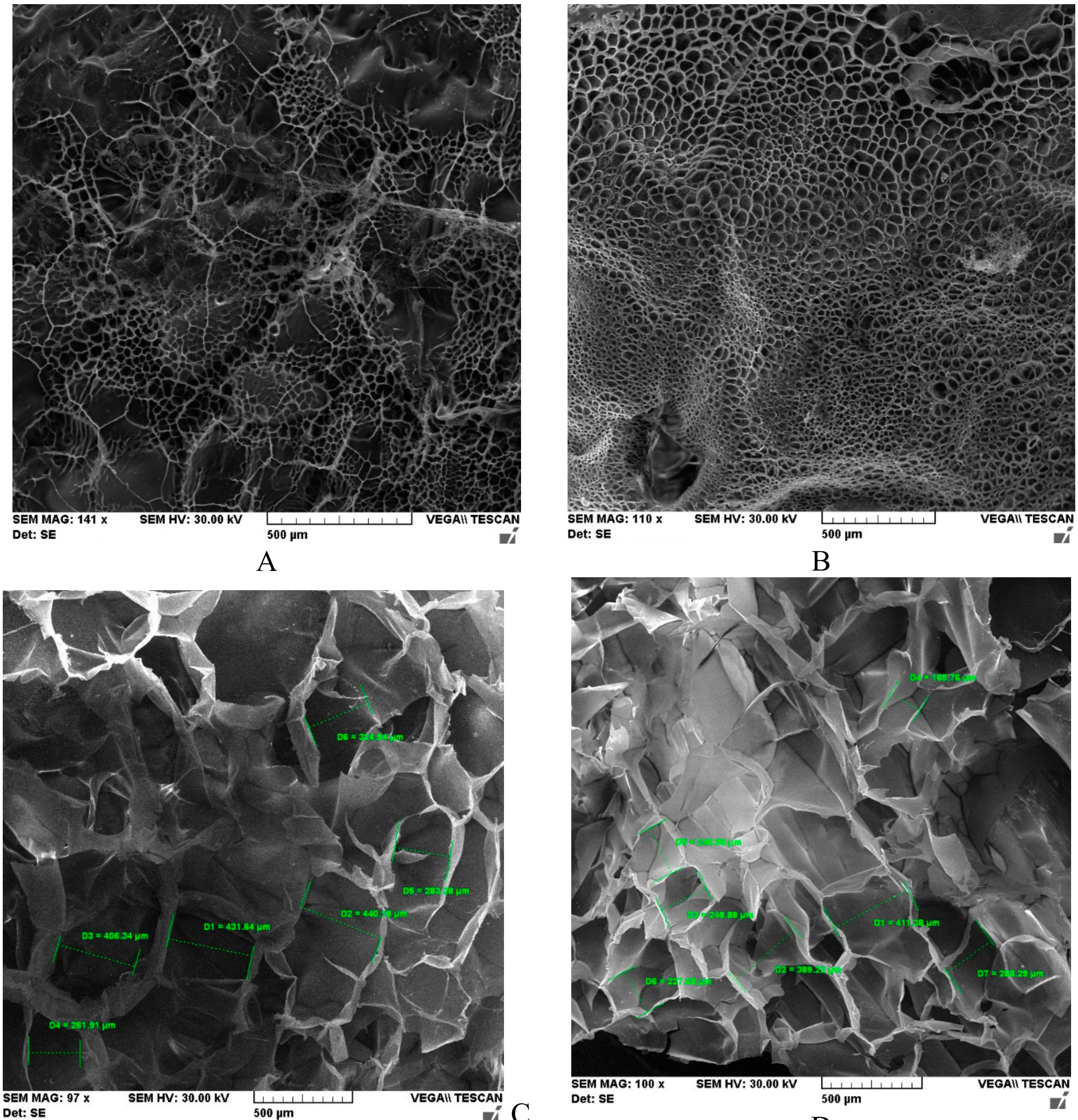

Figure S8. SEM images of lyophilized samples

(A) ***P1***, (B) ***P2***, (C) ***P2-TiO₂***, (D) ***P2-ZnO***

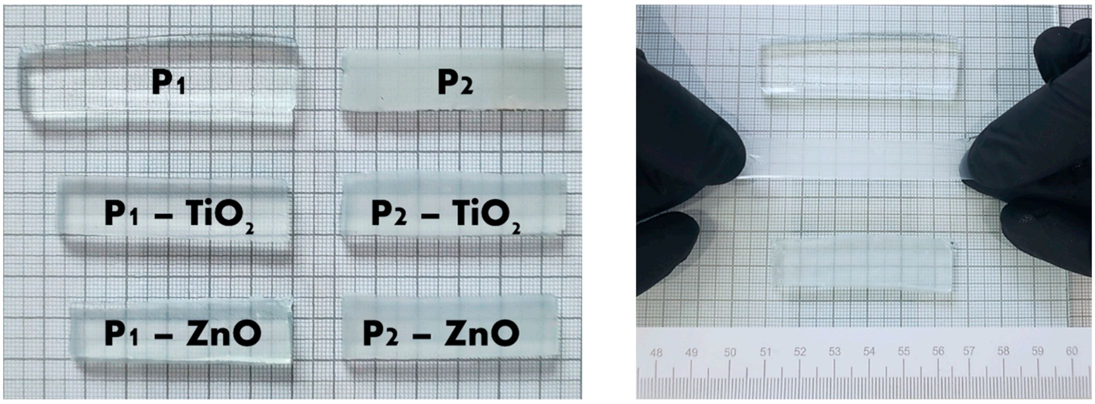

Figure S9. Exemplification of equilibrium - swollen nanocomposite hydrogel films

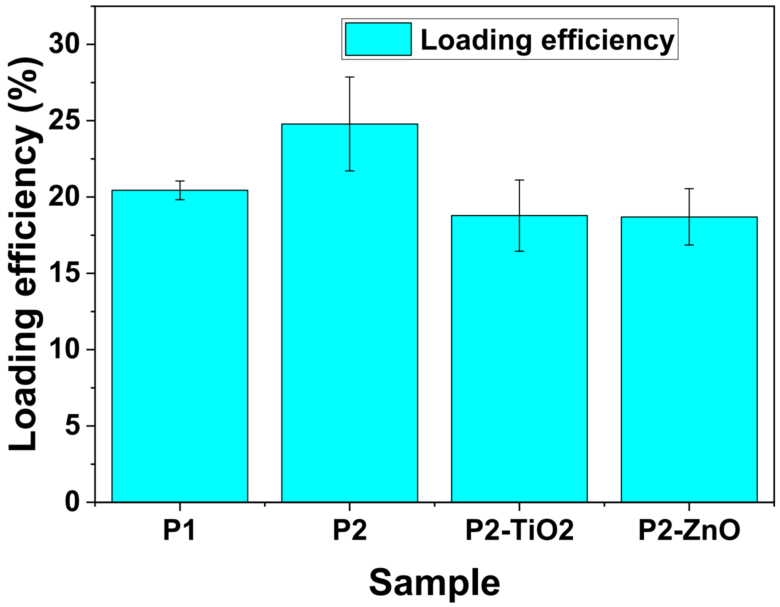

Figure S10. Nafcillin loading efficiency in the hydrogels

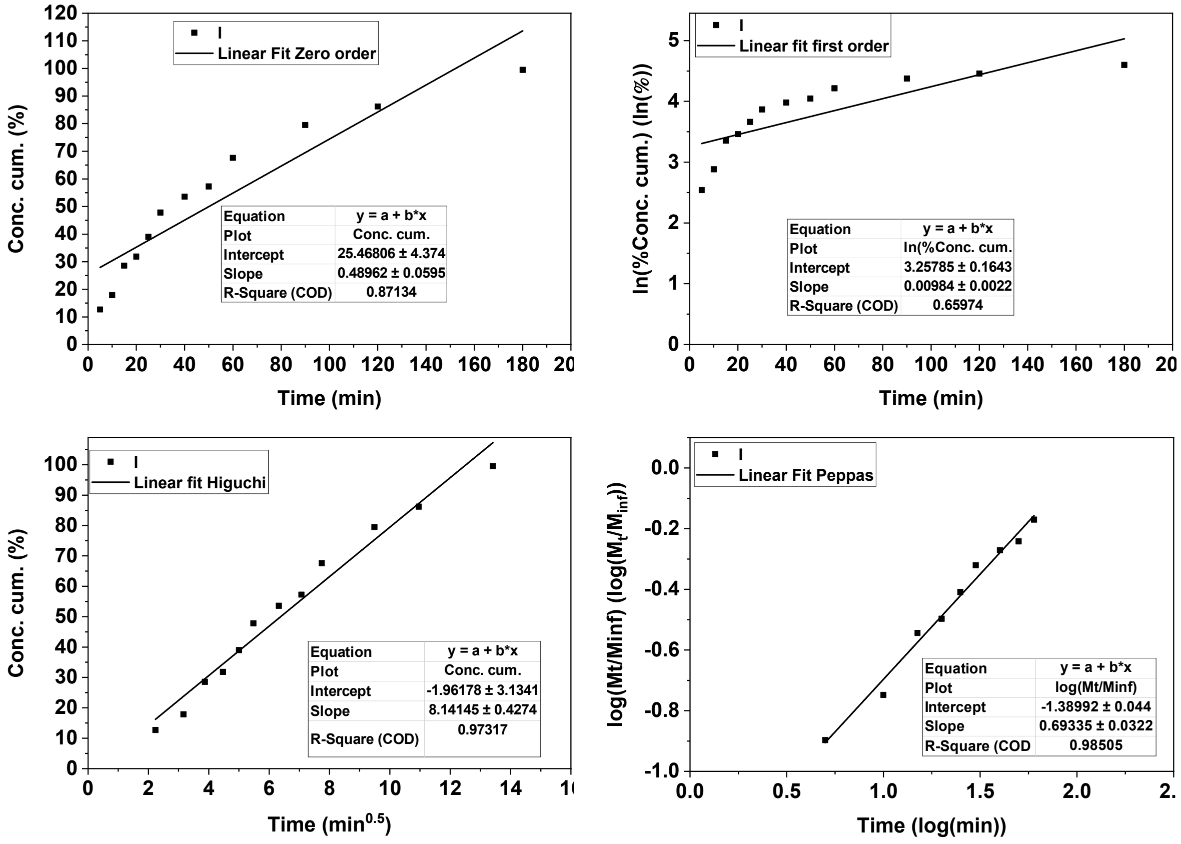

Figure S11. Nafcillin release - data fitting for sample P1

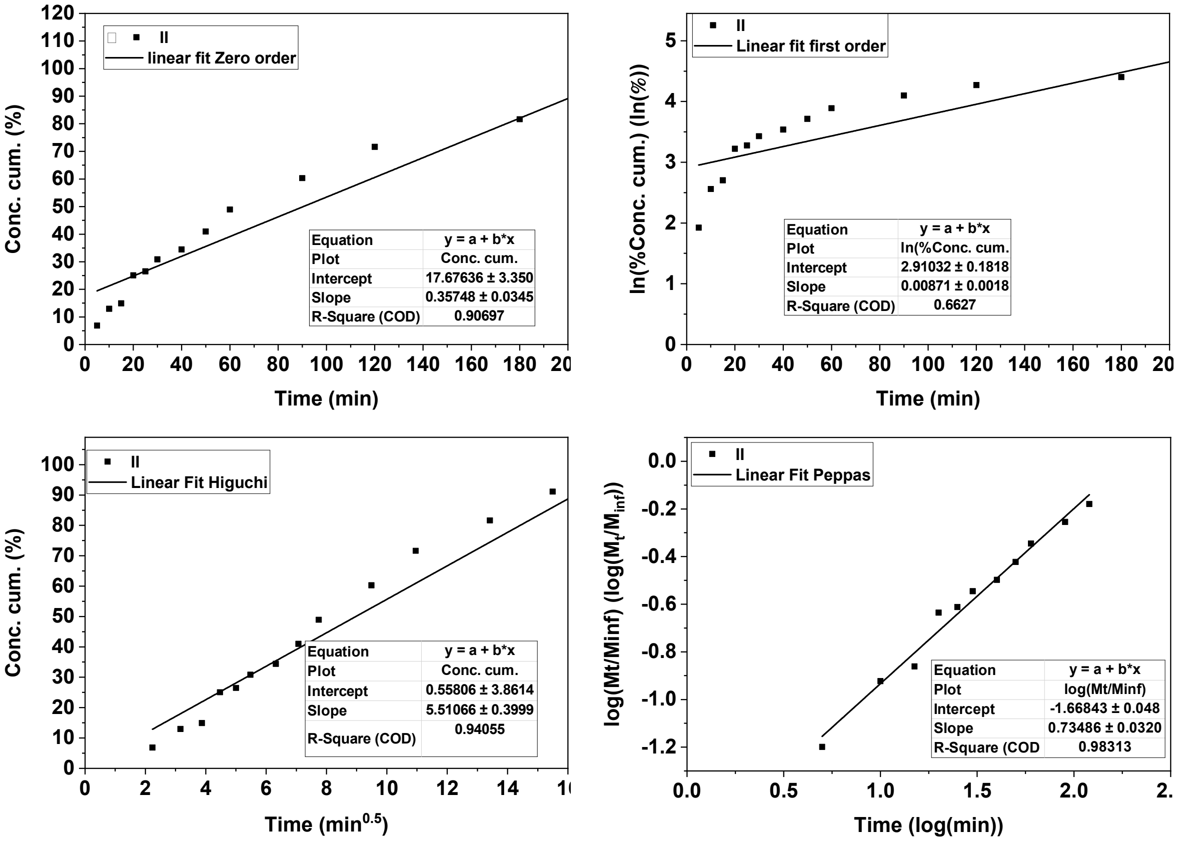

Figure S12. Nafcillin release - data fitting for sample P2

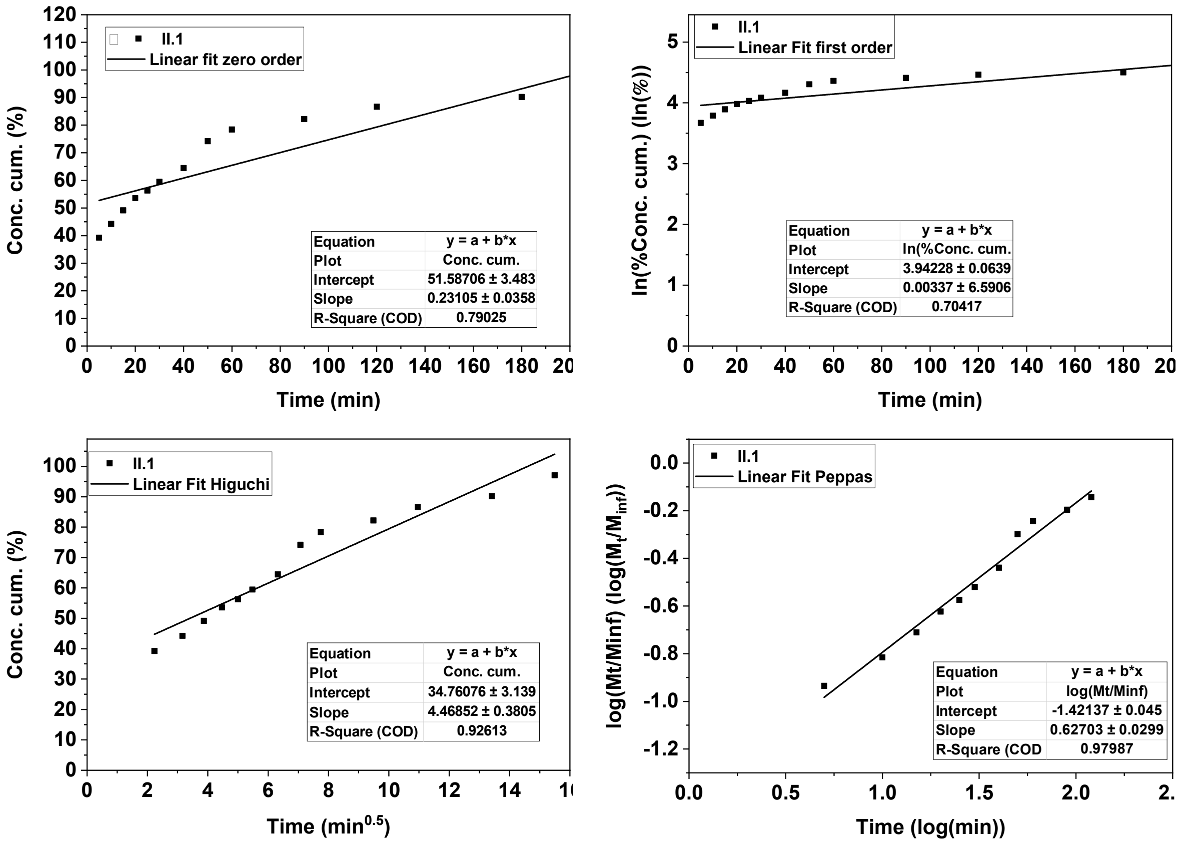

Figure S13. Nafcillin release - data fitting for sample P2.1 (P2 – TiO_2)

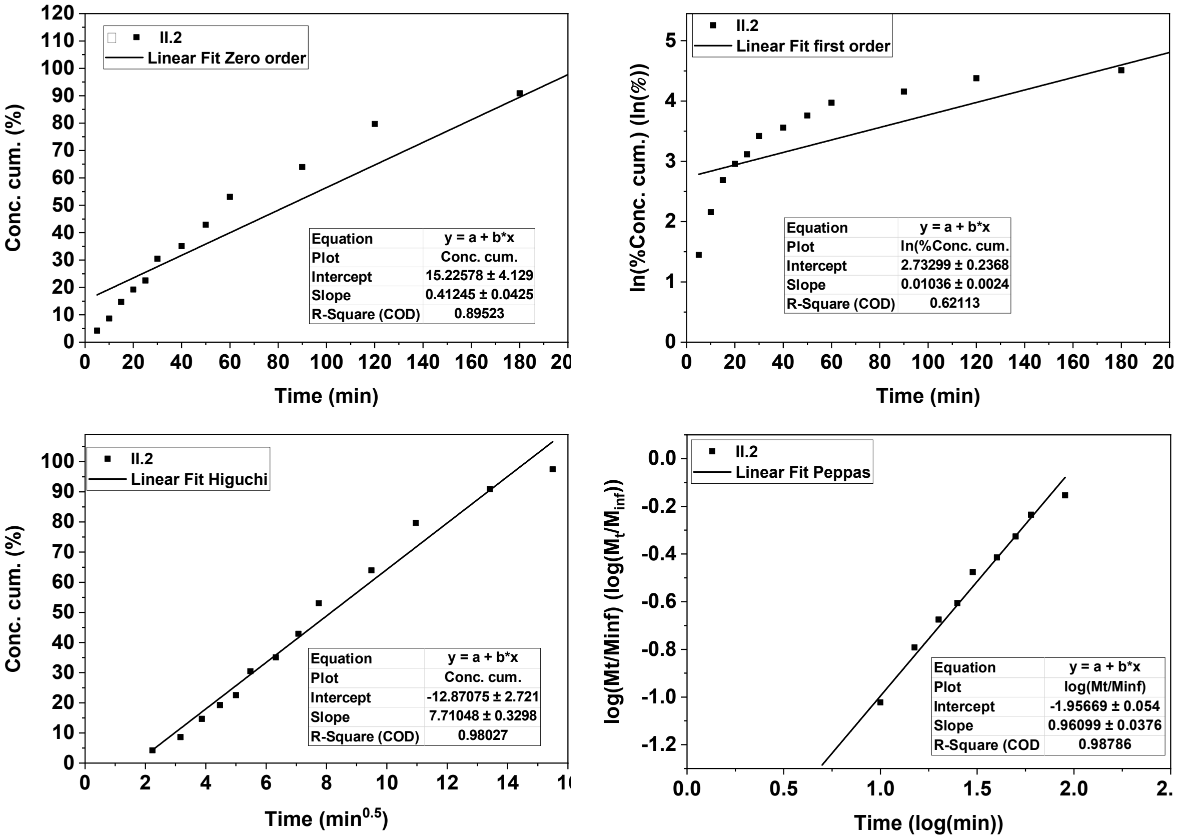

Figure S14 – Nafcillin release - data fitting for sample P2.2 (P2 - ZnO)
